# Functional trajectories before and after loss of ambulation in Duchenne muscular dystrophy and implications for clinical trials

**DOI:** 10.1371/journal.pone.0304099

**Published:** 2024-06-03

**Authors:** Craig M. McDonald, James Signorovitch, Eugenio Mercuri, Erik H. Niks, Brenda Wong, Mirko Fillbrunn, Gautam Sajeev, Erica Yim, Ibrahima Dieye, Debra Miller, Susan J. Ward, Nathalie Goemans, Investigators from the PRO-DMD-01 Study, Collaborative Trajectory Analysis Project (cTAP)

**Affiliations:** 1 Department of Physical Medicine and Rehabilitation and Department of Pediatrics, University of California Davis Health System, Sacramento, California, United States of America; 2 Analysis Group Inc., Boston, Massachusetts, United States of America; 3 Collaborative Trajectory Analysis Project, Cambridge, Massachusetts, United States of America; 4 Child Neurology Unit e Centro Nemo, IRCCS Fondazione Policlinico Gemelli, Università Cattolica del Sacro Cuore, Rome, Italy; 5 Department of Neurology, Leiden University Medical Center, Leiden, The Netherlands; 6 Department of Pediatrics and Neurology, University of Massachusetts Chan Medical School, Worcester, Massachusetts, United States of America; 7 CureDuchenne, Newport Beach, California, United States of America; 8 University Hospitals, Child Neurology, Leuven, Belgium; University of Minnesota Medical School, UNITED STATES

## Abstract

This study examined functional trajectories of subjects during the transition phase between ambulatory and non-ambulatory Duchenne muscular dystrophy (DMD) to inform clinical trial designs for new therapeutics. Ambulatory, pulmonary, and upper limb function leading up to loss of ambulation (LoA) and non-ambulatory measures following LoA were quantified; time ordering of pulmonary and upper limb milestones relative to LoA were determined; and the 10-second time threshold for 10-meter walk/run (10MWR) as a marker of approaching LOA was explored. Included in this analysis were 51 subjects aged between 7 and 18 years who experienced LoA during follow-up in the PRO-DMD-01 natural history study. Mean age at LoA was 12.7 (7.1–18.6) years. Mean annual rates of decline in forced vital capacity (FVC) <80%-predicted and performance of upper limb (PUL) 1.2 total score were smaller before than after LoA, but not significantly (FVC %-predicted: 5.6% vs. 10.1%, *p* = 0.21; PUL 1.2 total score: 2.3 vs. 3.8 units, *p* = 0.20). More than half of patients experienced clinically significant deficits in FVC %-predicted and PUL 1.2 before experiencing LoA. Among subjects with baseline 10MWR >10 s, those with <1 year to LoA had similar mean ages but significantly worse mean ambulatory function at baseline compared to those with ≥1 year to LoA. Enriching DMD clinical trials for patients with declining pulmonary or upper limb function is achievable without restricting enrollment to non-ambulatory patients. The sequencing of LoA and initial deficits in pulmonary and upper limb function varied across patients and highlights the potential for composite outcomes or multi-outcome trial designs to assess disease-modifying therapies more comprehensively.

## Introduction

Duchenne Muscular Dystrophy (DMD) is a rare, progressive, X-linked recessive disease with a worldwide prevalence of 4.8 per 100,000 individuals and 7.1 per 100,000 males [1, 2]. DMD is characterized by progressive muscle weakness and atrophy, eventually leading to loss of ambulation (LoA), usually in the early teenage years, followed by loss of upper limb function, cardiac and pulmonary dysfunction, and early mortality [3, 4]. Stages of disease progression can be defined by profiles of functional deficits through which almost all patients progress, albeit at different rates [5].

There is no cure for DMD, and treatments such as corticosteroids, beta-blockers, cardiac medications, non-invasive ventilation, physical and occupational therapy, gastrointestinal and nutritional support, contracture prevention strategies, and surgery for spine deformity are used to manage disease progression [6–9]. Safety and efficacy of novel therapeutics, including mutation-specific approaches such as exon skipping therapies and stop codon readthrough medications have been assessed in clinical trials over the last decade, with only a few receiving accelerated or conditional approvals [10, 11].

Recruitment for DMD clinical trials is challenging due to disease rarity, genetic variability, and the desire, amid heterogeneity in stages and rates of disease progression across patients, to enroll homogenous populations for the detection of treatment effects [12–17]. Multiple DMD clinical trials in the past decade have focused on the ambulant population and have measured drug effects on the 6-minute walk distance (6MWD) or other ambulatory motor function assessments [18–21]. Clinical trials with these outcomes have typically excluded patients thought likely to experience LoA during the trial period by imposing minimum functional thresholds at baseline [18, 19]. The rationale for this exclusion has been that LoA may be unavoidable or irreversible during a 1-year study period in those who have reached a critical threshold of weakness and are experiencing a precipitous decline in motor abilities, even with effective therapy. Including patients who experience LoA cannot complete the ambulatory assessments that contribute to the trial’s primary endpoint, and scores of non-ambulant patients may skew the assessment of ambulant patients.

Alongside therapeutic advances for ambulatory patients, the development of therapeutics for DMD patients in later stages of the disease is a priority [22, 23]. To this end, drug developers have evaluated treatment effects on pulmonary and upper limb function because these functions are the next to decline in patients who have started to lose ambulatory function. Many of the trials targeting these endpoints have required patients to be non-ambulatory at baseline, motivated in part by natural history data showing reliable declines in upper limb and pulmonary function among non-ambulant patients (NCT02286947) (NCT01009294). More recently, trials of pulmonary function [23] or other motor function assessments [24, 25] have enrolled patients regardless of baseline LoA status. However, such trial designs have been less common, and there remains a population of “gap patients” who are on the cusp of LoA and thus have been deemed ineligible for most DMD clinical trials.

To better represent the full spectrum of DMD disease stages in clinical trials, a quantitative understanding of functional trajectories during the transition phase between the late ambulatory and early non-ambulatory stages of DMD is needed. The present study aimed to: 1) quantify the trajectories of ambulatory, pulmonary, and upper limb function leading up to LoA and non-ambulatory measures following LoA; 2) determine time ordering of pulmonary and upper limb milestones relative to LoA; and 3) explore the 10-second completion time threshold for 10-meter walk/run (10MWR) as a prognostic marker of approaching LoA by quantifying time to LoA and other milestones following this threshold.

## Materials and methods

### Study subjects

This analysis included subjects who experienced LoA during follow-up in PRO-DMD-01 (NCT01753804), a prospective natural history study of disease progression among boys with genetically confirmed DMD.

Subjects were considered to have experienced LoA at the first study visit at which they were unable to walk 10 m unaided or had a 6MWD of 0 m, after being ambulatory at study entry (i.e., able to complete the 10MWR at the baseline study visit).

PRO-DMD-01 enrolled and followed 269 ambulatory and non-ambulatory boys at 16 care centers in North America, South America, and Europe from 2012 to 2016. Study visits occurred every 6 months. Follow-up ranged from 1 to 3 years. Ethics review boards at the participating centers approved the study protocol, and informed consent/assent was obtained from all participants and their legal representatives. Data from PRO-DMD-01 were provided for the present study by CureDuchenne [26], a 501(c)3 DMD patient foundation. The clinical co-authors Craig M. McDonald, Eugenio Mercuri, Erik H. Niks, Brenda Wong, and Nathalie Goemans were among the primary/co-investigators of the original PRO-DMD-01 study and had access to information that could identify individual participants at the time of data collection or afterwards.

### Study measures

Functional measures were recorded before LoA, at time of LoA, and after LoA.

### Ambulatory function

Ambulant assessments, including the 6MWD test (with accelerometry where available), timed function tests (i.e., 10MWR, stair climb, stair descend, and supine to stand), and North Star Ambulatory Assessment (NSAA) were conducted before LoA.

To characterize subjects’ function at the time of transition from ambulatory to non-ambulatory phase, performance on a revised Egen Klassifikation (EK2-R) scale at time of LoA was also summarized [27, 28]. The EK2-R is a clinical tool designed to assess functioning in non-ambulatory DMD. The EK2-R scale (S1 Table) is administered through an interview with the subject, asking about how 17 individual functions are normally performed, followed, if possible, by a demonstration of how they are performed. In this study, performance on individual functions was scored from 0–3 or 0–4, with higher scores indicating better function, and the EK2-R total score, calculated by summing scores across all functions, ranged from 0–53. An NSAA to EK2-R bridging item, which characterizes those whose performance falls between the least able on the NSAA and the most able on the EK2-R, was also summarized.

### Pulmonary function

Pulmonary function tests were conducted based on the American Thoracic Society criteria [29] with subjects encouraged to give their best effort. Forced vital capacity (FVC) was assessed from 3 acceptable and repeatable efforts following at least 3, but no more than 6 repetitions. FVC %-predicted was calculated based on the Hankinson equation [30].

### Upper limb function

Upper limb function was assessed using the performance of upper limb (PUL) scale version 1.2, which was designed by the PUL Physiotherapy Working Group to evaluate motor performance in the upper limb of individuals with dystrophinopathies [31, 32]. The PUL 1.2 entry item assesses a subject’s ability to lift their arms with and without holding a weighted cup, and the ability to hold a pen, pick up a coin, or drive a powered wheelchair. Specific combinations of these abilities are scored on a scale of 0 (no useful function) to 6 (can lift both arms with elbows fully extended and touch hands above head). A subject’s entry score guides the specific tasks evaluated in subsequent items. The PUL 1.2 has been recently validated in a test-retest trial including both ambulatory and non-ambulatory MD patients [33].

### Myometry

Muscle strength was assessed using a handheld microFET2 myometer on the dominant side only. Muscle groups evaluated included shoulder flexors and knee extensors. Measures of muscle strength (in kilograms) were standardized as a percentage of body weight (i.e., [muscle strength measured by myometry in kg / patient’s body weight in kg] x 100). Subjects were in the sitting position, which ensured continuity between ambulatory and non-ambulatory phases. Up to 3 tests were allowed, in addition to any practice, and the best results were recorded.

### Range of movement

Ankle contractures (dorsiflexion) were measured at baseline using a simple universal goniometer.

### Echocardiography

Echocardiography was not a required study measure in PRO-DMD-01. Rather, investigators were instructed to enter echocardiographic data into the case report forms if the assessment was already part of the clinical workflow. This analysis includes left ventricular ejection fraction (LVEF) outcomes among the subset of patients with data recorded.

### Disease milestones

Clinically meaningful functional thresholds were used to define disease milestones. FVC %-predicted <80% was used to represent the onset of clinically meaningful pulmonary decline, as this is defined as the threshold for mild restrictive lung disease [34]. A PUL entry item score <6, which represents less than full shoulder abduction, was used to represent the onset of upper limb deficits.

### Statistical analysis

Subject characteristics were summarized at the time of LoA, overall and stratified by age at LoA (≤12 years at LoA or >12 years at LoA, as the median age at LoA reported in natural history studies is approximately 12 years [35, 36], and this was also close to the median age at LoA in this study sample. Subject characteristics were compared across strata of age at LoA using chi-square tests for categorical variables and t-tests for continuous variables. Longitudinal trajectories of functional measures were described before and after LoA. Mean trajectories were estimated using longitudinal mixed effects models accounting for repeated measures and fitted to data over all available assessments during the pre- and post-LoA periods. Linear trajectories were investigated, and tests were conducted for differences in estimated slope before versus after LoA, using tests of the corresponding parameter in the mixed effects models. Both unadjusted and adjusted models were considered, with adjusted models accounting for age, steroid duration, and steroid type at the time of LoA. Results from unadjusted models are reported, as findings were similar for both models. Time to pulmonary and upper limb milestone events were estimated using Kaplan-Meier (KM) curves, with time rescaled relative to the timing of LoA. To study the relative order in which milestone events occurred, subjects with LoA were cross-classified based on whether they reached milestones before or after LoA (or were censored for milestones).

A potential marker for approaching LoA was explored in subjects at the first study visit with 10MWR >10 s; this threshold has been used as an exclusion criterion in some ambulatory clinical trials (NCT03907072), or as an inclusion criterion in late ambulatory to non-ambulatory trials [25]. Subject characteristics and NSAA item scores overall and stratified by time to LoA of <1 or ≥1 year, and time to pulmonary and upper limb milestones estimated using KM curves, were summarized for this subgroup. All statistical analyses were conducted using R (version 3.6.) [37].

## Results

### Patient characteristics at LoA

A total of 269 subjects were enrolled in PRO-DMD-01; of these, 219 subjects were ambulant and 50 subjects were non-ambulant at baseline. A total of 51 subjects first experienced LoA during study follow-up and met the criteria for inclusion in the present analysis. Characteristics of these 51 subjects at the time of LoA, overall and stratified by age at LoA (≤12 years at LoA [*n* = 21] or >12 years at LoA [*n* = 30]) are summarized in Table 1.

**Table 1:** Subject characteristics at LoA.

Demographics & vitals	All patients at LoA	LoA age ≤12 years	LoA age >12 years	*p* value
	*N* = 51	*n* = 21	*n* = 30	
Age (years)				<0.001 *
Mean ± SD	12.70 ± 2.87	9.99 ± 1.47	14.60 ± 1.91	
Median	12.48	10.14	14.47	
IQR	(10.81, 14.69)	(8.72, 11.13)	(12.89, 15.56)	
Range	(7.09, 18.64)	(7.09, 11.96)	(12.36, 18.64)	
Height (cm)	136.14 ± 9.77	134.76 ± 11.72	137.10 ± 8.22	0.41
Weight (kg)	41.19 ± 11.22	34.19 ± 9.51	46.09 ± 9.72	<0.001 *
BMI (kg/m^2^)	22.08 ± 5.21	18.62 ± 3.87	24.50 ± 4.67	<0.001 *
**Steroid exposure**				
**Steroid type**				
Deflazacort	32 (62.8%)	10 (47.6%)	22 (73.3%)	0.12
Prednisone	15 (29.4%)	9 (42.9%)	6 (20.0%)	
Missing / N (%)	4 / 51 (7.84%)	2 / 21 (9.52%)	2 / 30 (6.67%)	
Steroid duration (months)	62.09 ± 38.06	40.19 ± 22.53	77.42 ± 39.46	<0.001 *
**Steroid regimen**				<0.01
Once daily	37 (75.5)	10 (50.0)	26 (89.7)	
Other	12 (24.5)	10 (50.0)	3 (10.3)	
Missing	2/ 51 (3.9%)	1/21 (4.8%)	1/30 (3.3%)	
**Contractures (left or right ankle dorsiflexion >20 degrees, sitting)**				
Contracture	6 (12.8%)	5 (26.3%)	1 (3.6%)	0.03
No contracture	41 (87.2%)	14 (73.7%)	27 (96.4%)	
Missing / N (%)	4 / 51 (7.8%)	2 / 21 (9.5%)	2 / 30 (6.7%)	
**Upper limb function**				
PUL 1.2 total score				0.72
Mean ± SD	63.67 ± 5.39	63.32 ± 4.77	63.90 ± 5.83	
Median	64.5	63	65	
IQR	(61.00, 68.00)	(61.00, 66.50)	(61.00, 68.00)	
Range	(48.00, 73.00)	(54.00, 70.00)	(48.00, 73.00)	
Missing / N (%)	3 / 51 (5.88)	2 / 21 (9.52)	1 / 30 (3.33)	
**Pulmonary function**				
FVC (L)	1.83 ± 0.54	1.62 ± 0.57	1.97 ± 0.47	0.03 *
Missing / N (%)	8 / 51 (15.7%)	3 / 21 (14.3%)	5 / 30 (16.7%)	
FVC %-predicted	78.58 ± 19.45	70.17 ± 17.66	84.64 ± 18.69	0.01 *
FVC %-predicted <80% N (%)	21 (48.84%)	14 (77.78%)	7 (28.00%)	<0.01*
Missing / N (%)	8 / 51 (15.7%)	3 / 21 (14.3%)	5 / 30 (16.7%)	
**Myometry (standardized to body weight)**				
Shoulder flexor	0.10 ± 0.09	0.10 ± 0.06	0.11 ± 0.10	0.71
Knee extensor	0.07 ± 0.05	0.05 ± 0.03	0.08 ± 0.06	0.04 *
**Cardiac function**				
LVEF (%)	58.71 ± 4.53	60.85 ± 2.68	57.00 ± 5.24	0.23
Missing / N (%)	42 / 51 (82.3%)	17 / 21 (81.0%)	25 / 30 (83.3%)	
**Selected Egen Klassifikation items**				
Ability to stand item score				0.79
0: Unable to be stood	4 (9.30%)	3 (15.79%)	1 (4.17%)	
1: Able to stand with full body support	11 (25.58%)	5 (26.32%)	6 (25.00%)	
2: Able to stand with knees and hips supported, as when using standing aids	6 (13.95%)	2 (10.53%)	4 (16.67%)	
3: Able to stand with knees supported, as when using braces	9 (20.93%)	4 (21.05%)	5 (20.83%)	
4: Able to stand independently	13 (30.23%)	5 (26.32%)	8 (33.33%)	
Missing / N (%)	8 / 51 (15.69%)	2 / 21 (9.52%)	6 / 30 (20.00%)	
Physical well-being (related to respiratory insufficiency)				<0.05 *
0: Palpitations and perspiring in addition to weight loss, appetite loss, and poor sleep	0 (0.00%)	0 (0.00%)	0 (0.00%)	
1: Has loss of weight, loss of appetite, and associated poor sleep	0 (0.00%)	0 (0.00%)	0 (0.00%)	
2: Easily tires, has difficulty resting in a chair or in bed	5 (11.36%)	5 (26.32%)	0 (0.00%)	
3: No complaints, feels good (no daytime tiredness)	39 (88.64%)	14 (73.68%)	25 (100.00%)	
Missing / N (%)	7 / 51 (13.73%)	2 / 21 (9.52%)	5 / 30 (16.67%)	
Daytime fatigue item score				0.10
0: Get tired during day even with rest and limited activity	0 (0.00%)	0 (0.00%)	0 (0.00%)	
1: Need to limit activity and have a rest period to avoid getting too tired	3 (6.82%)	3 (15.79%)	0 (0.00%)	
2: Need to limit activity to avoid getting too tired	13 (29.55%)	6 (31.58%)	7 (28.00%)	
3: Doesn’t get tired during day.	28 (63.64%)	10 (52.63%)	18 (72.00%)	
Missing / N (%)	7 / 51 (13.73%)	2 / 21 (9.52%)	5 / 30 (16.67%)	

BMI, body mass index; IQR, interquartile range; FVC, forced vital capacity; LoA, loss of ambulation; LVEF, left ventricular ejection fraction; PUL, performance of upper limb; SD, standard deviation.

Among all 51 subjects, at the time of LoA, mean age was 12.7 years (range, 7.1–18.6 years), mean FVC %-predicted was 78.6%, mean PUL 1.2 total score was 64, and 13% of the subjects had ankle contractures. In subjects ≤12 years at LoA and >12 years at LoA, respectively, mean height was 134.8 cm and 137.1 cm, mean body weight was 34.2 kg and 46.1 kg, and mean body mass index was 18.6 kg/m^2^ and 24.5 kg/m^2^. Steroid use at time of LoA also differed between the 2 groups: compared to subjects >12 years at LoA, subjects ≤12 years at LoA were less likely to be on a daily steroid regimen (50% vs. 89.7%) and had shorter mean duration of steroid treatment (40.2 months vs. 77.4 months).

**Fig 1:**
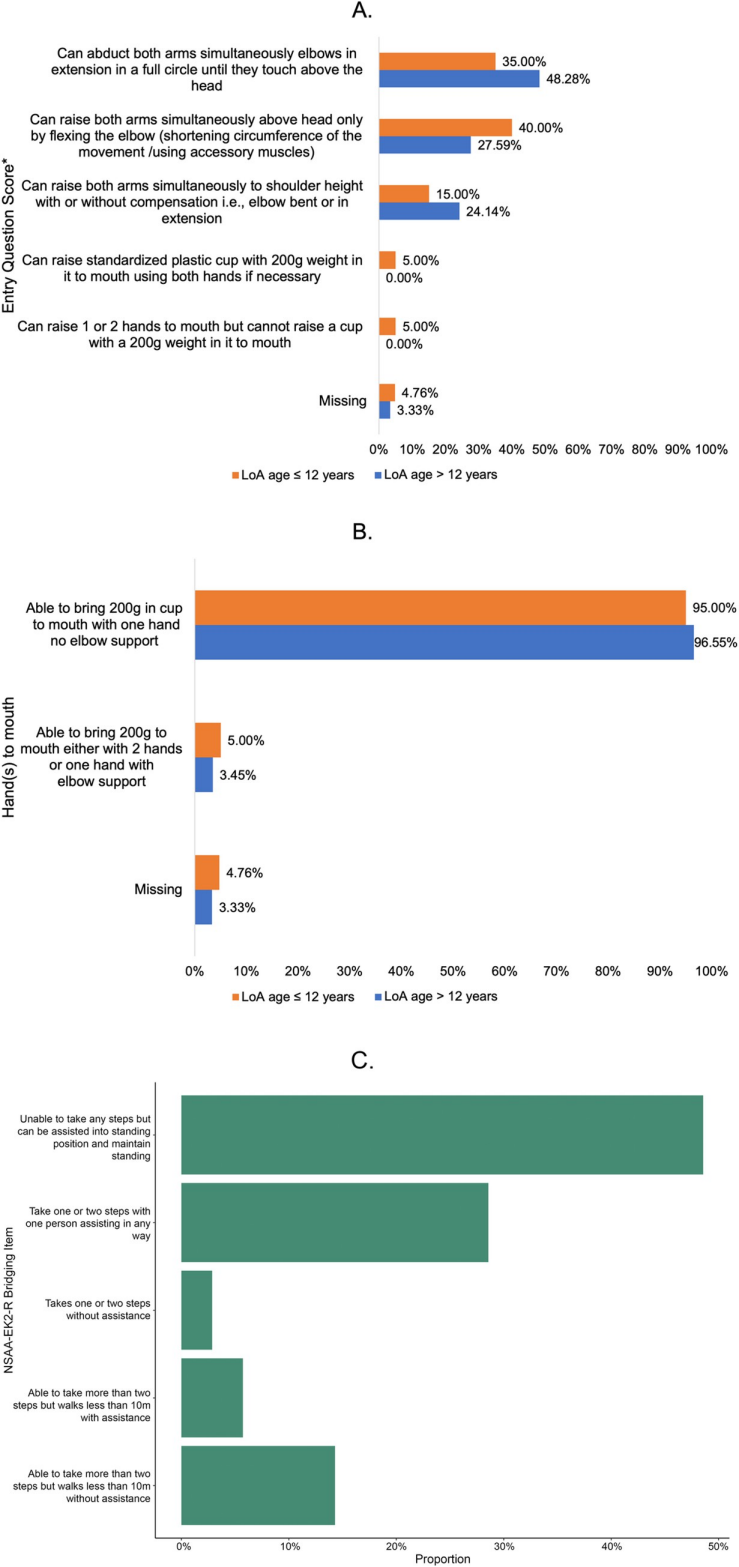
**A.** Performance upper limb (PUL) assessments at LoA–entry questions. LoA, loss of ambulation. **B.** Performance upper limb (PUL) assessments at LoA–hand(s)-to-mouth questions. LoA, loss of ambulation. **C.** NSAA-EK2-R bridging item. EK2-R, revised Egen Klassifikation; NSAA, North Star Ambulatory Assessment.

Subjects ≤12 years at LoA had significantly lower mean FVC (1.62 vs. 1.97 L) and FVC %-predicted (70.17% vs. 84.64%), and were more likely to have already reached the clinical milestone of FVC %-predicted <80% than subjects >12 years at LoA. Subjects ≤12 years at LoA had significantly lower mean knee extensor strength (5% vs. 7%) and a significantly higher rate of ankle contractures (26.3% vs. 3.6%). There were no apparent differences in mean PUL 1.2 scores (63.3 vs. 63.9), mean shoulder flexor strength (10% vs. 11%), or LVEF (approximately 60% for both groups) between subjects >12 years or ≤12 years at LoA. Responses to PUL 1.2 assessments stratified by age at LoA are presented in Fig 1.

At the time of LoA, the majority of subjects (77.1%) were unable to take any steps without assistance, while a few subjects were able to take smaller numbers of steps (less than 10 meters) with or without assistance (based on the NSAA-EK2 bridging item, Fig 1C, S1 Table). Most subjects were also able to stand with some type of assistance (90.7%, Table 1), use hands and arms for eating without elbow support (88.6%) and fully cough without help (88.6%) at the time of LoA (S1 Table). However, these abilities did not differ significantly between those with younger vs. older ages at LoA. Subjects with ≤12 years at LoA were significantly more likely to report needing rest during the day (26.3% vs. 0.0%, *p*<0.05) compared to those >12 years at LoA. The need to adapt activities to daytime fatigue was higher among subjects with ≤12 years at LoA (47.4% vs. 28.0%) but not significantly different (*p* = 0.1).

### Trajectories of functional assessments before and after LoA

**Fig 2:**
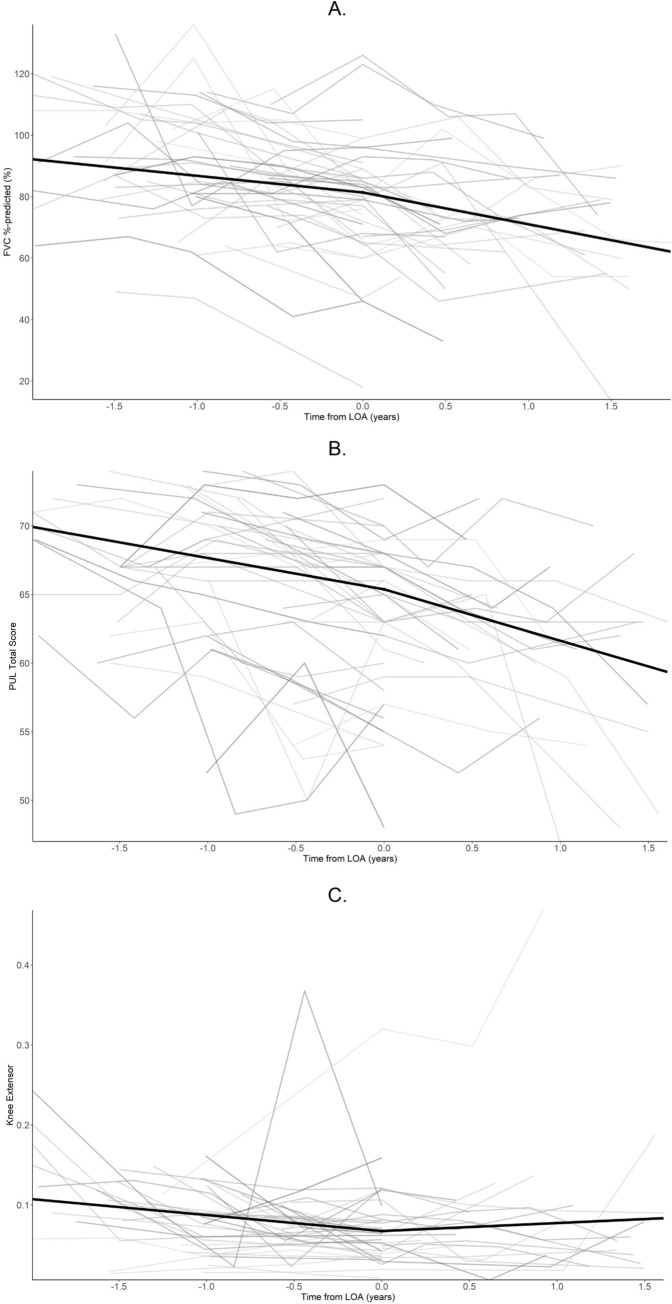
**A.** FVC %-predicted trajectories before and after LoA. FVC, forced vital capacity; LoA, loss of ambulation. **B.** PUL 1.2 total score trajectories before and after LoA. LoA, loss of ambulation; PUL, performance of upper limb. **C.** Knee extensor strength, as a proportion of body weight, before and after LoA. LoA, loss of ambulation.

Longitudinal trajectories of functional assessments before and after LoA are shown in Fig 2A–2C. Within-patient rates of change in FVC %-predicted and the PUL 1.2 total scores were visually highly variable from one visit to the next.

Mean annual decline for FVC %-predicted was 5.6% (standard error: 2.1%) percentage points before LoA and 10.1% (2.2%) after LoA (Fig 2A). Mean annual decline for PUL 1.2 total score was 2.3 (0.7) units before LoA and 3.8 (0.8) units after LoA (Fig 2B). Mean rates of decline in FVC %-predicted and PUL 1.2 total score before and after LoA were not statistically significantly different.

Mean knee extensor strength exceeded 10% of body weight at 2 years prior to LoA, had a mean annual decline of 3% until LoA, and was approximately 6% of body weight during the 1.5 years after LoA (Fig 2C). Mean rate of decline in knee extensor strength before and after LoA was significantly different *(p*<0.01). However, the mean change in knee extensor strength estimated for the post-LoA period appears to be heavily influenced by outlying values for 2 patients (see S1 Fig excluding outlier subjects); these results should therefore be interpreted with caution.

### Timing of pulmonary and upper limb milestones relative to LoA

**Fig 3:**
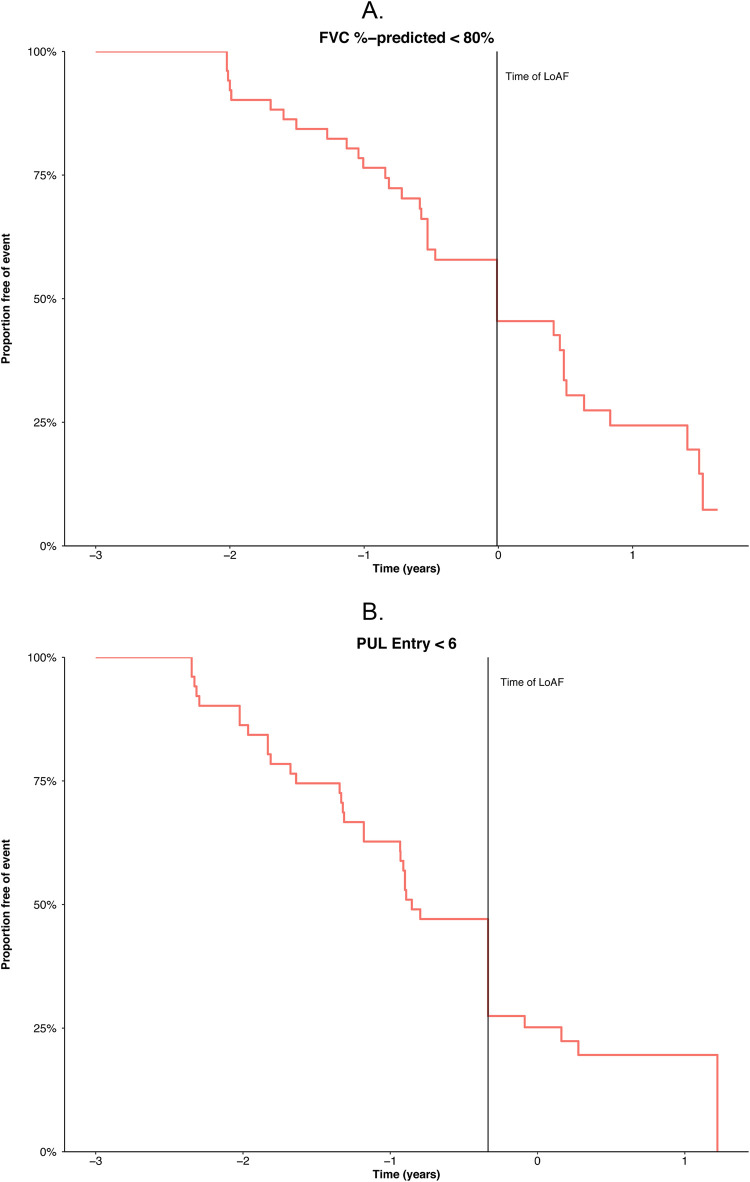
**A.** Time to FVC %-predicted <80%, relative to LoA. FVC, forced vital capacity. **B.** Time to deficit on the PUL entry question, relative to LoA. LoA, loss of ambulation; PUL, performance of upper limb.

The timing of pulmonary and upper limb milestones relative to LoA is shown in Fig 3A and 3B. Starting approximately 2 years before LoA, the proportion of subjects who experienced FVC %-predicted <80% increased by approximately 25% per year (Fig 3A). At the time of LoA, approximately 50% of subjects had experienced FVC %-predicted <80%, and the remainder reached this milestone within 2 years after LoA. The proportion of patients with PUL 1.2 entry score <6 started increasing approximately 2 years before LoA and then increased slightly more rapidly. At the time of LoA, approximately 75% of subjects had a PUL 1.2 entry score <6, and the remainder reached this milestone within 1.5 years after LoA (Fig 3B). In 55% of patients, both FVC <80%-predicted and PUL 1.2 entry score <6 had the same temporal relationship to LoA (i.e., both thresholds occurred before, or with, or after LoA) (Table 2).

**Table 2:** Timing of pulmonary and upper limb milestones relative to LoA.

		PUL Entry Question <6			
		Before LoA	At LoA	After LoA*	Total
**FVC %-predicted <80%**	**Before LoA**	**15 (29.4%)**	4 (7.8%)	2 (3.9%)	21 (41.2%)
	**At LoA**	1 (2%)	**3 (5.9%)**	2 (3.9%)	6 (11.8%)
	**After LoA** *	11 (21.6%)	3 (5.9%)	**10 (19.6%)**	24 (47.1%)
	**Total**	27 (52.9%)	10 (19.6%)	14 (27.5%)	51 (100%)

*Includes patients who were not observed to reach the milestone while under follow-up in the study.

FVC, forced vital capacity; LoA, loss of ambulation, PUL, performance of upper limb.

### Outcomes and baseline characteristics in patients with 10MWR >10 s

**Fig 4:**
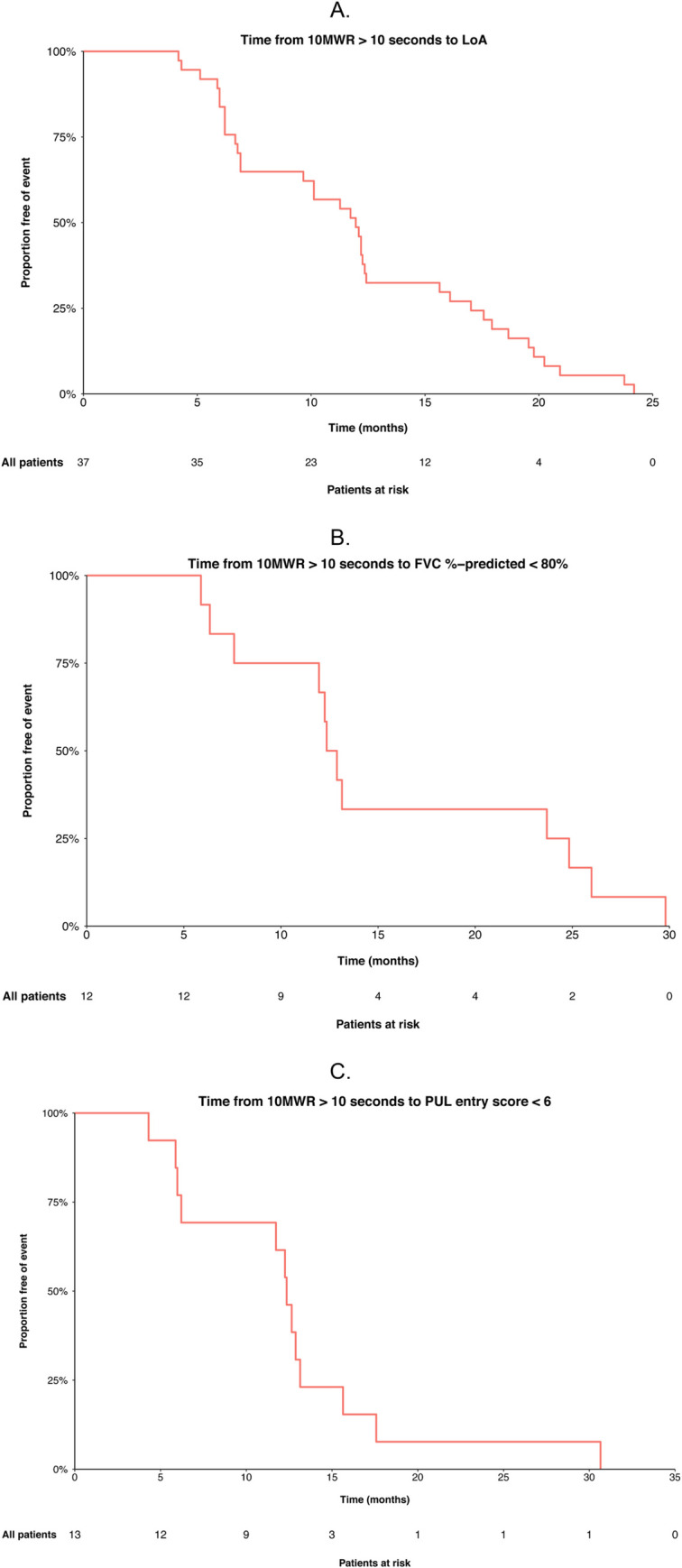
**A.** Time to LoA from first assessment with 10MWR exceeding 10 s. 10MWR, 10-meter walk/run; LoA, loss of ambulation. **B.** Time to FVC %-predicted <80% from first assessment with 10MWR exceeding 10 s. 10MWR, 10-meter walk/run; FVC, forced vital capacity; s, seconds. **C.** Time to PUL question <6 from first assessment with 10MWR exceeding 10 s. 10MWR, 10-meter walk/run; PUL, performance of upper limb; s, seconds.

Time to LoA from first visit with 10MWR >10 s is shown in Fig 4A–4C. A total of 37 subjects had a first visit with 10MWR >10 s (Table 3). Median time to LoA from the first visit with 10MWR >10 s was 1 year, and all subjects experienced LoA within 2 years (Fig 4A). Subjects with shorter (<1 year) vs. longer (≥1 year) time to LoA had significantly worse ambulatory function at their first visit with 10MWR >10 s, as indicated by longer average 10MWR (14.38 vs. 11.17 s, respectively), shorter 6MWD (163.5 m vs. 224.8 m, respectively), a lower proportion able to climb 4 stairs (42.1% vs. 82.4%, respectively), and longer times to climb 4 stairs among those who were able (22.4 s vs. 12.2 s). When comparing subjects with shorter versus longer time to LoA, no significant differences were observed in age, FVC or FVC %-predicted, PUL total score, myometry, hand to mouth ability, or LVEF (Table 3).

**Table 3:** Patient characteristics at the first assessment with 10MWR exceeding 10 s.

Demographics & vitals	Number of patients	Time to LoA <1 year	Time to LoA ≥1 year	*p* value
	*N* = 37	*n =* 19	*n* = 18	
Age (years)				0.72
Mean ± SD	11.67 ± 3.12	11.49 ± 2.96	11.86 ± 3.35	
Median	11.51	11.72	11.43	
IQR	(9.46, 13.83)	(9.53, 13.50)	(9.00, 13.75)	
Range	(6.57, 17.15)	(6.57, 16.47)	(7.13, 17.15)	
Missing / N (%)	0 / 37 (0.00)	0 / 19 (0.00)	0 / 18 (0.00)	
Height (cm)	131.43 ± 10.33	132.18 ± 8.98	130.68 ± 11.74	0.67
Weight (kg)	36.39 ± 11.39	36.96 ± 12.31	35.81 ± 10.71	0.77
BMI (kg/m^2^)	20.68 ± 4.74	20.72 ± 5.05	20.64 ± 4.55	0.96
**Steroid exposure**				
Steroid type				
Deflazacort	24 (64.86%)	11 (57.89%)	13 (72.22%)	0.57
Prednisone	12 (32.43%)	7 (36.84%)	5 (27.78%)	0.81
Steroid duration (months)	56.54 ± 36.99	55.47 ± 33.79	57.67 ± 41.06	0.86
**Steroid regimen**				0.82
Once daily	24 (64.9)	12 (63.1)	12 (66.7)	
Other	13 (35.1)	7 (36.9)	6 (33.3)	
Missing	0/ 37 (0.0%)	0/19 (0.0%)	0/18 (0.0%)	
**Ambulatory function**				
Timed 10MWR (s)	12.82 ± 3.79	14.38 ± 4.69	11.17 ± 1.22	0.01 *
6MWD (meters)	194.99 ± 60.44	163.47 ± 59.05	224.76 ± 45.75	<0.001 *
Able to perform rise from supine	12 (34.29%)	5 (26.32%)	7 (43.75%)	0.47
Timed rise from supine (s)	25.58 ± 17.10	26.57 ± 10.32	24.81 ± 21.61	0.85
Able to perform 4-stair climb	22 (61.11%)	8 (42.11%)	14 (82.35%)	0.02 *
Timed 4-stair climb (s)	16.74 ± 10.62	22.41 ± 11.63	12.20 ± 7.32	0.01 *
NSAA total score	9.83 ± 3.18	8.39 ± 2.64	11.28 ± 3.06	<0.001 *
**Upper limb**				
PUL 1.2 total score				0.89
Mean ± SD	64.64 ± 6.24	64.78 ± 6.68	64.47 ± 5.90	
Median	66	66.5	66	
IQR	(61.00, 69.00)	(61.50, 70.00)	(61.00, 68.50)	
Range	(49.00, 74.00)	(49.00, 74.00)	(52.00, 73.00)	
Missing / N (%)	4 / 37 (10.81)	1 / 19 (5.26)	3 / 18 (16.67)	
** Entry question**				0.7
Can raise standardized plastic cup with 200 g weight in it to mouth using both hands if necessary	1 (2.94%)	1 (5.26%)	0 (0.00%)	
Can raise both arms simultaneously to shoulder height with or without compensation i.e., elbow bent or in extension	1 (2.94%)	0 (0.00%)	1 (6.67%)	
Can raise both arms simultaneously above head only by flexing the elbow (shortening circumference of the movement/using accessory muscles)	16 (47.06%)	8 (42.11%)	8 (53.33%)	
Can abduct both arms simultaneously, elbows in extension in a full circle, until they touch above the head	16 (47.06%)	10 (52.63%)	6 (40.00%)	
Missing / N (%)	3 / 37 (8.11%)	0 / 19 (0.00%)	3 / 18 (16.67%)	
Hand(s) to mouth				0.44
Able to bring 200 g in cup to mouth either with 2 hands or 1 hand with elbow support	1 (2.94%)	0 (0.00%)	1 (6.67%)	
Able to bring 200 g in cup to mouth with 1 hand no elbow support	33 (97.06%)	19 (100.00%)	14 (93.33%)	
Missing / N (%)	3 / 37 (8.11%)	0 / 19 (0.00%)	3 / 18 (16.67%)	
**Pulmonary function**				
FVC (L)	1.78 ± 0.57	1.72 ± 0.54	1.85 ± 0.61	0.52
FVC %-predicted (%)	88.03 ± 21.45	84.83 ± 16.10	91.87 ± 26.61	0.36
FVC %-predicted <80% N (%)	11 (33.33%)	7 (38.89%)	4 (26.67%)	0.71
**Myometry (standardized to body weight)**				
Shoulder flexor	0.10 ± 0.05	0.10 ± 0.05	0.10 ± 0.04	0.57
Knee extensor	0.08 ± 0.04	0.08 ± 0.04	0.08 ± 0.04	0.70
**Cardiac function**				
LVEF (%)	62.08 ± 2.46	62.50 ± 3.08	61.55 ± 1.68	0.6

6MWD, 6-minute walking distance; 10MWR, 10-meter walk/run; BMI, body mass index; IQR, interquartile range; FVC, forced vital capacity; LoA, loss of ambulation; LVEF, left ventricular ejection fraction; NSAA, North Star Ambulatory Assessment; PUL, performance of upper limb; s, seconds; SD, standard deviation.

**Fig 5:**
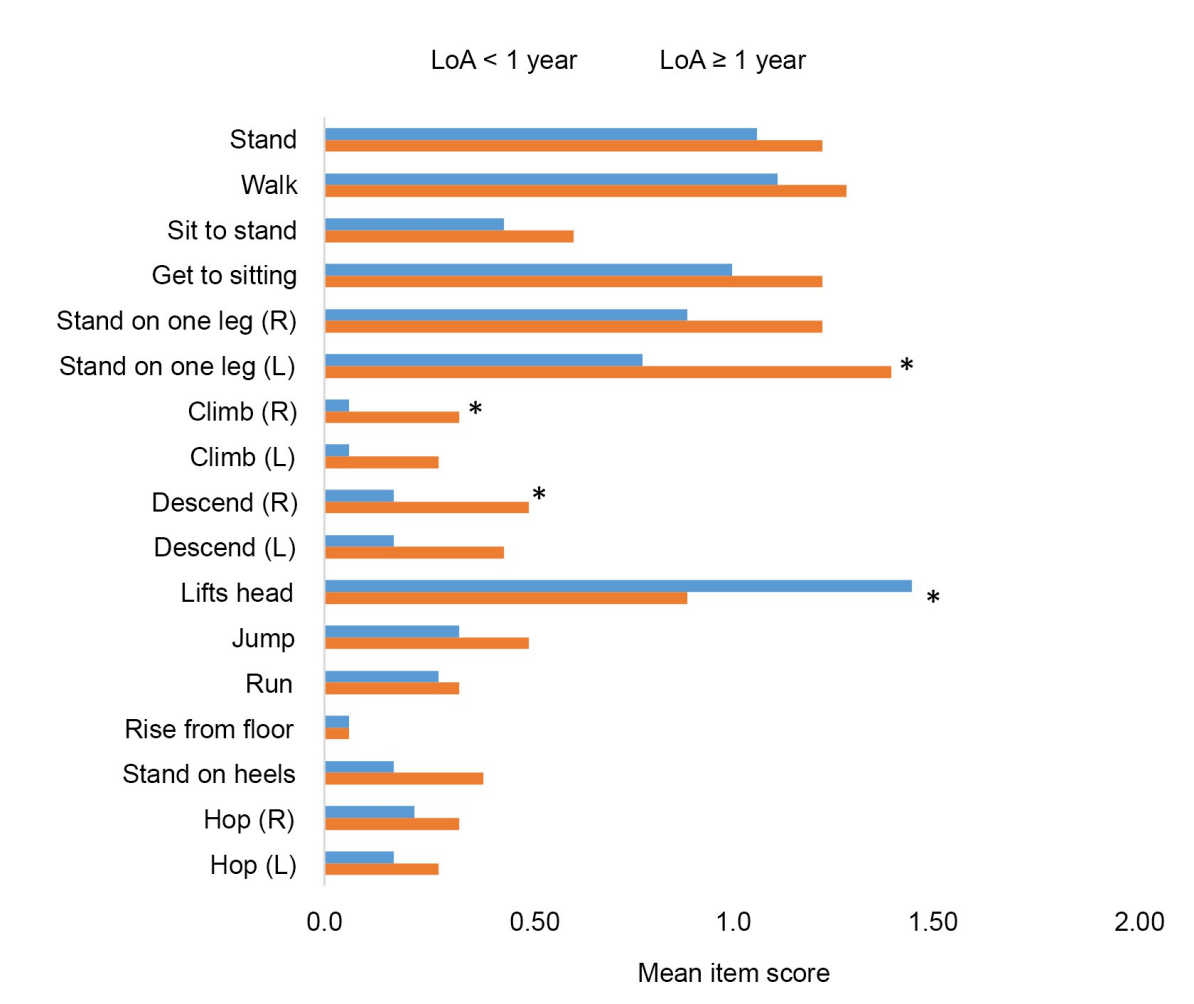
Mean NSAA scores at first assessment with 10MWR exceeding 10s, stratified by time to LoA. 10MWR, 10-meter walk/run; LoA, loss of ambulation; NSAA, North Star Ambulatory Assessment; s, seconds.

Mean NSAA item scores at the time of first visit with 10MWR >10 s stratified by time to LoA are shown in Fig 5. Mean NSAA item scores were generally lower (worse) for subjects with shorter time to LoA than for those with longer time to LoA. Subjects with shorter time to LoA demonstrated significantly worse function for the standing on 1 leg (left) and climbing and descending a box step (right) items (Fig 4A–4C). There was inconsistency in the lifts head item with other NSAA items in the 19 subjects with time to LoA <1 year and unexpected preservation of the lifts head item (mean score 1.5 with a mean total NSAA of only 8.39 ± 2.64) and significantly better function in the lifts head item in those within 1 year of LoA vs. ≥1 year to LoA (Fig 5). Among subjects with 10MWR >10 s and FVC %-predicted ≥80% at the first visit (*n* = 12), median time to FVC %-predicted <80% was approximately 1 year, and all subjects reached this milestone within 2.5 years (Fig 4B). Among subjects with 10MWR >10 and PUL 1.2 entry item 6 at the first visit (*n =* 13), median time to PUL 1.2 entry item <6 was approximately 1 year, and all subjects reached this milestone within 2.5 years (Fig 4C).

## Discussion

This study characterized the transition phase between ambulatory and non-ambulatory DMD to better understand how these “patients in transition” can be included in clinical trials of new therapeutics. Ambulatory, pulmonary, and upper limb trajectories were quantified in 51 subjects aged between 7 and 18 years during the transition from ambulatory to non-ambulatory DMD and following LoA. Findings showed that more than half of subjects experienced clinically significant deficits in FVC %-predicted and PUL 1.2 before experiencing LoA.

These findings have implications for inclusion criteria in clinical trials seeking to investigate treatment effects on pulmonary and upper limb function in subjects with DMD. In the present study, LoA did not necessarily predict early declines in FVC %-predicted or the PUL 1.2, while remaining ambulatory did not preclude having early declines in these measures. This implies that clinical trials should not require patients to be non-ambulatory, to enrich for patients experiencing early declines on these measures. Rather, enrollment should be based on functional thresholds for pulmonary or upper limb functions in broad populations that include ambulatory and non-ambulatory subjects. In the present study, we also observed that interpatient variability in rates of change in FVC %-predicted and the PUL 1.2 total scores were visually higher than the mean change in these measures, highlighting the need to better understand prognostic factors for changes in pulmonary and upper limb function.

The present study revealed no evidence of a strictly consistent ordering in which subjects reached the clinical milestones FVC %-predicted <80% (clinically meaningful pulmonary decline) [34] or a PUL 1.2 entry-item score <6 (onset of upper limb deficits) relative to LoA, and lower extremity contractures are not expected to impact FVC and PUL assessments. In contrast, more consistent ordering has been observed for purely ambulatory milestones such as loss of ability to rise from floor, climb stairs, walk, and stand [5, 16]. Heterogeneous ordering of milestones across functional domains and individual subjects suggests that composite endpoints for clinical trials, particularly composite time-to-event endpoints (e.g., time to first clinically meaningful progression in any functional domain) could be helpful for measuring drug effects. Such composite outcomes would be sensitive to treatment effects across functional domains and would address the heterogeneity in rates and timing of progression across patients and domains that is characteristic of DMD. Efforts to develop and validate composite outcomes are warranted, especially given the growing need to study long-term milestones across DMD disease stages in open-label extension or post-market studies of treatments receiving conditional or accelerated approvals in DMD.

Patient characteristics at the time of LoA differed between subjects who lost ambulation at earlier age (≤12 years) vs. later age (>12 years). Subjects who lost ambulation later, despite being on average approximately 4.5 years older at the time of LoA, were approximately the same height but weighed significantly more than subjects who lost ambulation earlier. The shorter-than-expected height and greater weight in these subjects who lost ambulation later may be explained by their more prolonged steroid use: subjects who lost ambulation later were more likely to be on a daily steroid regimen and had longer duration of steroid use at the time of LoA. Consistent with prior research, pulmonary function was more preserved at the time of LoA in subjects who lost ambulation later [38]. Differences in daytime fatigue are consistent with the lower average FVC %-predicted observed among the subjects who lost ambulation early. Upper limb function did not differ at the time of LoA between patients who lost ambulation earlier versus later.

Findings from the present study suggest that 10MWR >10 s has utility as an indicator of an approaching LoA milestone, defined as being within 24 months of loss of ambulation. Consistent with previous reports, subjects crossing this threshold had a median time to LoA of 1 year, and all reached LoA within 2 years [39, 40]. Notably, there was no association between age at which subjects crossed this 10MWR threshold and subsequent time to LoA. However, there were significant associations between multiple indicators of poor ambulatory function and time to LoA. Interestingly, the mean NSAA total score for patients <1 year to LoA was 8.39 ± 2.64, and for patients 1 to 2 years from LoA, it was 11.28 ± 3.06. This is consistent with a mean NSAA raw score of 9 among subjects who were 1 year from LoA in a study of over 500 patients followed longitudinally by the UK NorthStar Network [12].

Subjects who crossed the 10MWR threshold but had not yet experienced FVC %-predicted <80% or PUL total score <6 had median times to these events of approximately 1 year. This timeframe may be suitable for studying drug effects, potentially in event-driven trial designs. Composite outcomes based on reaching either of these milestones and for patients who have already crossed these thresholds, experiencing further clinically significant declines in FVC %-predicted or PUL total score should be explored.

One evaluation not helpful to LoA prognostication was the lifts head item of the NSAA, for which better function was associated with shorter time to LoA after reaching 10MWR >10 s. This observation is perhaps unsurprising, since the lifts head item of the NSAA is not consistent with the sequential disease severity construct of the NSAA based on RASCH analyses [41].

Strengths of this study include the use of prospectively collected natural history data from a multicenter study PRO-DMD-01 and the ability to select patients who have experienced LoA on study with rigorous functional assessments before and after the event. However, small sample sizes for LoA events limited statistical power to detect associations and our ability to further investigate other factors, such as dystrophin genotypes or steroid regimen, that might impact outcomes around the time of LoA. Attempts to understand if rate of change in upper limb and pulmonary assessments differs pre- and post-loss of ambulation were also limited by substantial variability.

## Conclusions

There is heterogeneity across patients in age at LoA and in the ordering and magnitude of deficits in pulmonary and upper limb function before and after LoA. Enriching trials for patients with declining pulmonary or upper limb function is achievable without restricting eligibility to non-ambulatory patients; this is particularly important when outcomes of interest evaluate parameters unrelated to lower extremity function such as pulmonary and upper limb function endpoints. Design of composite outcomes, or multi-outcome trial designs, which are sensitive to changes in multiple functional domains before and after LoA, should be investigated further.

## Supporting information

S1 Table NSAA-EK2-R bridging item and Egen Klassification at time of LoA. EK2-R, revised Egen Klassifikation; IQR, interquartile range; LoA, loss of ambulation; NSAA, North Star Ambulatory Assessment; SD, standard deviation.
(DOCX)

S1 Fig Knee extensor strength, as a proportion of body weight, before and after LoA (excluding outlier subjects). LoA, loss of ambulation.
(TIF)
