# Supplementary material for: Functional trajectories before and after loss of ambulation in Duchenne muscular dystrophy and implications for clinical trials
Source: PLoS One. 2024 Jun 3;19(6):e0304099. doi: 10.1371/journal.pone.0304099 (PMC11146704; doi:10.1371/journal.pone.0304099)
Supplement: S1 Table — EK2-R, revised Egen Klassifikation; IQR, interquartile range; LoA, loss of ambulation; NSAA, North Star Ambulatory Assessment; SD, standard deviation. (DOCX) [file pone.0304099.s001.docx]

**Appendix**

**Table 1. NSAA-EK2-R Bridging Item and Egen Klassification at time of LoA.**

|  | **Number of patients**  **N = 51** | **LoA before or at 12 years**  **N = 21** | **LoA after 12 years**  **N = 30** | **P-value** |
| --- | --- | --- | --- | --- |
| **NSAA-EK2-R Bridging Item** |  |  |  |  |
| Unable to take any steps but can be assisted into standing position and maintain standing | 17 (48.57%) | 6 (46.15%) | 11 (50.00%) | 0.73 |
| Take one or two steps with one person assisting in any way | 10 (28.57%) | 4 (30.77%) | 6 (27.27%) |  |
| Takes one or two steps without assistance | 1 (2.86%) | 0 (0.00%) | 1 (4.55%) |  |
| Able to take more than two steps but walks less than 10m with assistance | 2 (5.71%) | 0 (0.00%) | 2 (9.09%) |  |
| Able to take more than two steps but walks less than 10m without assistance | 5 (14.29%) | 3 (23.08%) | 2 (9.09%) |  |
| Missing / N (%) | 16 / 51 (31.37%) | 8 / 21 (38.10%) | 8 / 30 (26.67%) |  |
| **Egen Klassifikation** |  |  |  |  |
| **Egen Klassifikation Total Score** |  |  |  | 0.89 |
| Mean ± SD | 47.17 ± 3.37 | 47.33 ± 3.61 | 47.08 ± 3.40 |  |
| Median | 46 | 47.5 | 46 |  |
| IQR | (45.00, 50.75) | (45.25, 50.50) | (45.00, 50.25) |  |
| Range | (42.00, 52.00) | (42.00, 51.00) | (42.00, 52.00) |  |
| Missing / N (%) | 33 / 51 (64.71%) | 15 / 21 (71.43%) | 18 / 30 (60.00%) |  |
| **Egen Klassification Individual Items** |  |  |  |  |
| **Ability to use wheelchair** |  |  |  | 0.4 |
| 0: Uses power wheelchair but occasionally has difficulty steering | 0 (0.00%) | 0 (0.00%) | 0 (0.00%) |  |
| 1: Unable to use manual wheelchair, requires power wheelchair | 7 (17.07%) | 2 (12.50%) | 5 (20.00%) |  |
| 2: Able to use a manual wheelchair on flat ground, 10 meters > 1 minute | 1 (2.44%) | 1 (6.25%) | 0 (0.00%) |  |
| 3: Able to use a manual wheelchair on flat ground, 10 meters < 1 minute | 33 (80.49%) | 13 (81.25%) | 20 (80.00%) |  |
| Missing / N (%) | 10 / 51 (19.61%) | 5 / 21 (23.81%) | 5 / 30 (16.67%) |  |
| **Ability to transfer from wheelchair** |  |  |  | 0.49 |
| 0: Needs to be lifted with support of head when transferring from wheelchair | 0 (0.00%) | 0 (0.00%) | 0 (0.00%) |  |
| 1: Needs assistance to transfer with or without additional aids (hoist, easy glide) | 19 (45.24%) | 8 (47.06%) | 11 (44.00%) |  |
| 2: Able to transfer independently from wheelchair, with use of aids | 7 (16.67%) | 4 (23.53%) | 3 (12.00%) |  |
| 3: Able to transfer from wheelchair without help | 16 (38.10%) | 5 (29.41%) | 11 (44.00%) |  |
| Missing / N (%) | 9 / 51 (17.65%) | 4 / 21 (19.05%) | 5 / 30 (16.67%) |  |
| **Ability to stand** |  |  |  | 0.79 |
| 0: Unable to be stood | 4 (9.30%) | 3 (15.79%) | 1 (4.17%) |  |
| 1: Able to stand with full body support | 11 (25.58%) | 5 (26.32%) | 6 (25.00%) |  |
| 2: Able to stand with knees and hips supported, as when using standing aids | 6 (13.95%) | 2 (10.53%) | 4 (16.67%) |  |
| 3:.Able to stand with knees supported, as when using braces | 9 (20.93%) | 4 (21.05%) | 5 (20.83%) |  |
| 4: Able to stand independently | 13 (30.23%) | 5 (26.32%) | 8 (33.33%) |  |
| Missing / N (%) | 8 / 51 (15.69%) | 2 / 21 (9.52%) | 6 / 30 (20.00%) |  |
| **Ability to balance in the wheelchair** |  |  |  | < 0.05 * |
| 0: Unable to change positions of the upper part of the body, cannot sit without total support of the trunk and head. | 0 (0.00%) | 0 (0.00%) | 0 (0.00%) |  |
| 1: Able to move the upper part of the body <30 degrees from one side to the other | 3 (7.14%) | 3 (17.65%) | 0 (0.00%) |  |
| 2: Able to move the upper part of the body ≥30 degrees in all directions from the upright position, but cannot push himself upright | 2 (4.76%) | 1 (5.88%) | 1 (4.00%) |  |
| 3: Able to push himself upright from complete forward flexion by pushing up with hands | 6 (14.29%) | 4 (23.53%) | 2 (8.00%) |  |
| 4: Able to push himself upright from complete forward flexion without using arms | 31 (73.81%) | 9 (52.94%) | 22 (88.00%) |  |
| Missing / N (%) | 9 / 51 (17.65%) | 4 / 21 (19.05%) | 5 / 30 (16.67%) |  |
| **Ability to move the arms** |  |  |  | 0.07 |
| 0: Unable to move the hands against gravity but able to use the fingers | 0 (0.00%) | 0 (0.00%) | 0 (0.00%) |  |
| 1: Unable to lift the forearms against gravity but able to use the hands against gravity when the forearm is supported | 0 (0.00%) | 0 (0.00%) | 0 (0.00%) |  |
| 2: Unable to lift the arms above the head but able to raise the forearms against gravity, i.e., hand to mouth with/without elbow support | 3 (6.82%) | 3 (15.79%) | 0 (0.00%) |  |
| 3: Able to raise the arms above the head with or without compensatory movements. | 41 (93.18%) | 16 (84.21%) | 25 (100.00%) |  |
| Missing / N (%) | 7 / 51 (13.73%) | 2 / 21 (9.52%) | 5 / 30 (16.67%) |  |
| **Ability to use the hands and arms for eating** |  |  |  | 0.64 |
| 0: Has to be fed | 0 (0.00%) | 0 (0.00%) | 0 (0.00%) |  |
| 1: Eats and drinks with elbow support; with reinforcement of the opposite hand + or - aids | 0 (0.00%) | 0 (0.00%) | 0 (0.00%) |  |
| 2: Eats or drinks with support at elbow | 5 (11.36%) | 3 (15.79%) | 2 (8.00%) |  |
| 3: Able to eat and drink without elbow support | 39 (88.64%) | 16 (84.21%) | 23 (92.00%) |  |
| Missing / N (%) | 7 / 51 (13.73%) | 2 / 21 (9.52%) | 5 / 30 (16.67%) |  |
| **Ability to turn in bed** |  |  |  | 0.54 |
| 0: Unable to turn himself in bed. Has to be turned ≥ 4 times during the night | 1 (2.27%) | 1 (5.26%) | 0 (0.00%) |  |
| 1: Unable to turn himself in bed. Has to be turned 0-3 times during the night. | 1 (2.27%) | 0 0.00%%) | 1 4.00%) |  |
| 2: Can turn in some directions in bed or needs help to turn in bed (Needs rail to pull on, someone else needs to position legs, covers). | 3 (6.82%) | 2 (10.53%) | 1 (4.00%) |  |
| 3: Able to turn himself in bed with bedclothes. | 39 (88.64%) | 16 (84.21%) | 23 (92.00%) |  |
| Missing / N (%) | 7 / 51 (13.73%) | 2 / 21 (9.52%) | 5 / 30 (16.67%) |  |
| **Ability to cough** |  |  |  | 0.64 |
| 0: Unable to cough, needs suction and/or hyperventilation techniques or IPPB in order to keep airways clear | 0 (0.00%) | 0 (0.00%) | 0 (0.00%) |  |
| 1: Always needs help with coughing | 0 (0.00%) | 0 (0.00%) | 0 (0.00%) |  |
| 2: Has difficulty to cough but able to clear throat | 5 (11.36%) | 3 (15.79%) | 2 (8.00%) |  |
| 3: Able to cough effectively | 39 (88.64%) | 16 (84.21%) | 23 (92.00%) |  |
| Missing / N (%) | 7 / 51 (13.73%) | 2 / 21 (9.52%) | 5 / 30 (16.67%) |  |
| **Ability to speak** |  |  |  | 1 |
| 0: Speech is difficult to understand except to close relatives | 0 (0.00%) | 0 (0.00%) | 0 (0.00%) |  |
| 1: Speaks with a quiet voice and/or needs a breath after just a few words. | 0 (0.00%) | 0 (0.00%) | 0 (0.00%) |  |
| 2: Speaks normally, but cannot raise voice | 0 (0.00%) | 0 (0.00%) | 0 (0.00%) |  |
| 3: Powerful speech, able to sing and speak loudly | 44 (100.00%) | 19 (100.00%) | 25 (100.00%) |  |
| Missing / N (%) | 7 / 51 (13.73%) | 2 / 21 (9.52%) | 5 / 30 (16.67%) |  |
| **Physical well-being** |  |  |  | < 0.05 * |
| 0: Palpitations and perspiring in addition to weight loss, appetite loss and poor sleep | 0 (0.00%) | 0 (0.00%) | 0 (0.00%) |  |
| 1: Has loss of weight, loss of appetite and associated poor sleep | 0 (0.00%) | 0 (0.00%) | 0 (0.00%) |  |
| 2: Easily tires, has difficulty resting in a chair or in bed | 5 (11.36%) | 5 (26.32%) | 0 (0.00%) |  |
| 3: No complaints, feels good (no daytime tiredness). | 39 (88.64%) | 14 (73.68%) | 25 (100.00%) |  |
| Missing / N (%) | 7 / 51 (13.73%) | 2 / 21 (9.52%) | 5 / 30 (16.67%) |  |
| **Daytime fatigue** |  |  |  | 0.1 |
| 0: Get tired during day even with rest and limited activity | 0 (0.00%) | 0 (0.00%) | 0 (0.00%) |  |
| 1: Need to limit activity and have a rest period to avoid getting too tired | 3 (6.82%) | 3 (15.79%) | 0 (0.00%) |  |
| 2: Need to limit activity to avoid getting too tired | 13 (29.55%) | 6 (31.58%) | 7 (28.00%) |  |
| 3: Doesn’t get tired during day. | 28 (63.64%) | 10 (52.63%) | 18 (72.00%) |  |
| Missing / N (%) | 7 / 51 (13.73%) | 2 / 21 (9.52%) | 5 / 30 (16.67%) |  |
| **Head control** |  |  |  | 1 |
| 0: When sitting still in a wheelchair, needs head support | 0 (0.00%) | 0 (0.00%) | 0 (0.00%) |  |
| 1: Needs head support when driving wheelchair | 0 (0.00%) | 0 (0.00%) | 0 (0.00%) |  |
| 2: Needs head support when going up and down slope (15 degree standard ramp) | 0 (0.00%) | 0 (0.00%) | 0 (0.00%) |  |
| 3: Does not need head support. | 40 (100.00%) | 17 (100.00%) | 23 (100.00%) |  |
| Missing / N (%) | 11 / 51 (21.57%) | 4 / 21 (19.05%) | 7 / 30 (23.33%) |  |
| **Ability to control joystick** |  |  |  | 0.14 |
| 0: Unable to operate wheelchair. Needs another person to operate it. | 3 (11.54%) | 0 (0.00%) | 3 (18.75%) |  |
| 1: Uses other techniques for steering than joystick such as blowing sucking systems or scanned driving. | 0 (0.00%) | 0 (0.00%) | 0 (0.00%) |  |
| 2: Uses an adapted joystick or has adjusted wheelchair in order to use joystick. | 1 (3.85%) | 1 (10.00%) | 0 (0.00%) |  |
| 3: Uses a standard joystick without special adaptation | 22 (84.62%) | 9 (90.00%) | 13 (81.25%) |  |
| Missing / N (%) | 25 / 51 (49.02%) | 11 / 21 (52.38%) | 14 / 30 (46.67%) |  |
| **Food textures** |  |  |  | 0.41 |
| 0: Minimal oral intake | 0 (0.00%) | 0 (0.00%) | 0 (0.00%) |  |
| 1: Eats minced/pureed food | 1 (2.27%) | 1 (5.26%) | 0 (0.00%) |  |
| 2: Eats cut up or small pieces of food or avoids hard/chewy foods | 7 (15.91%) | 2 (10.53%) | 5 (20.00%) |  |
| 3: Eats all textures of food | 36 (81.82%) | 16 (84.21%) | 20 (80.00%) |  |
| Missing / N (%) | 7 / 51 (13.73%) | 2 / 21 (9.52%) | 5 / 30 (16.67%) |  |
| **Eating a meal** |  |  |  | 0.76 |
| 0: Unable to consume a whole meal even with additional time, assistance | 0 (0.00%) | 0 (0.00%) | 0 (0.00%) |  |
| 1: Able to consume a whole meal but requires substantially more time than others eating the same meal (15 m or more extra) or reduces portion size | 1 (2.38%) | 0 (0.00%) | 1 (4.17%) |  |
| 2: Able to consume a whole meal in the same time as others only with encouragement or needs some additional time (approx. 10 min) | 3 (7.14%) | 2 (11.11%) | 1 (4.17%) |  |
| 3: Able to consume a whole meal in the same time as others sharing the meal | 38 (90.48%) | 16 (88.89%) | 22 (91.67%) |  |
| Missing / N (%) | 9 / 51 (17.65%) | 3 / 21 (14.29%) | 6 / 30 (20.00%) |  |
| **Swallowing** |  |  |  | 1 |
| 0: Has trouble swallowing saliva or secretions. | 0 (0.00%) | 0 (0.00%) | 0 (0.00%) |  |
| 1: Has regular trouble swallowing food/drink or chokes on food/drink (more than once a month) | 0 (0.00%) | 0 (0.00%) | 0 (0.00%) |  |
| 2: May experience occasional (less than once a month) problems swallowing certain type of food or occasionally chokes | 3 (6.82%) | 1 (5.26%) | 2 (8.00%) |  |
| 3: Doesn’t choke or have problems swallowing when eating and drinking | 41 (93.18%) | 18 (94.74%) | 23 (92.00%) |  |
| Missing / N (%) | 7 / 51 (13.73%) | 2 / 21 (9.52%) | 5 / 30 (16.67%) |  |
| **Hand function** |  |  |  | 0.36 |
| 0: Cannot use hands | 0 (0.00%) | 0 (0.00%) | 0 (0.00%) |  |
| 1: Can write signature or send text or use remote control | 0 (0.00%) | 0 (0.00%) | 0 (0.00%) |  |
| 2: Can write two lines or use computer keyboard. | 21 (47.73%) | 11 (57.89%) | 10 (40.00%) |  |
| 3: Can unscrew the lid of a water or fizzy drink bottle and break the seal | 23 (52.27%) | 8 (42.11%) | 15 (60.00%) |  |
| Missing / N (%) | 7 / 51 (13.73%) | 2 / 21 (9.52%) | 5 / 30 (16.67%) |  |

EK2-R, revised Egen Klassifikation; IQR, interquartile range; LoA, loss of ambulation; NSAA, North Star Ambulatory Assessment; SD, standard deviation.
