# Supplementary figures and images for: Functional trajectories before and after loss of ambulation in Duchenne muscular dystrophy and implications for clinical trials
Source: PLoS One. 2024 Jun 3;19(6):e0304099. doi: 10.1371/journal.pone.0304099 (PMC11146704; doi:10.1371/journal.pone.0304099)

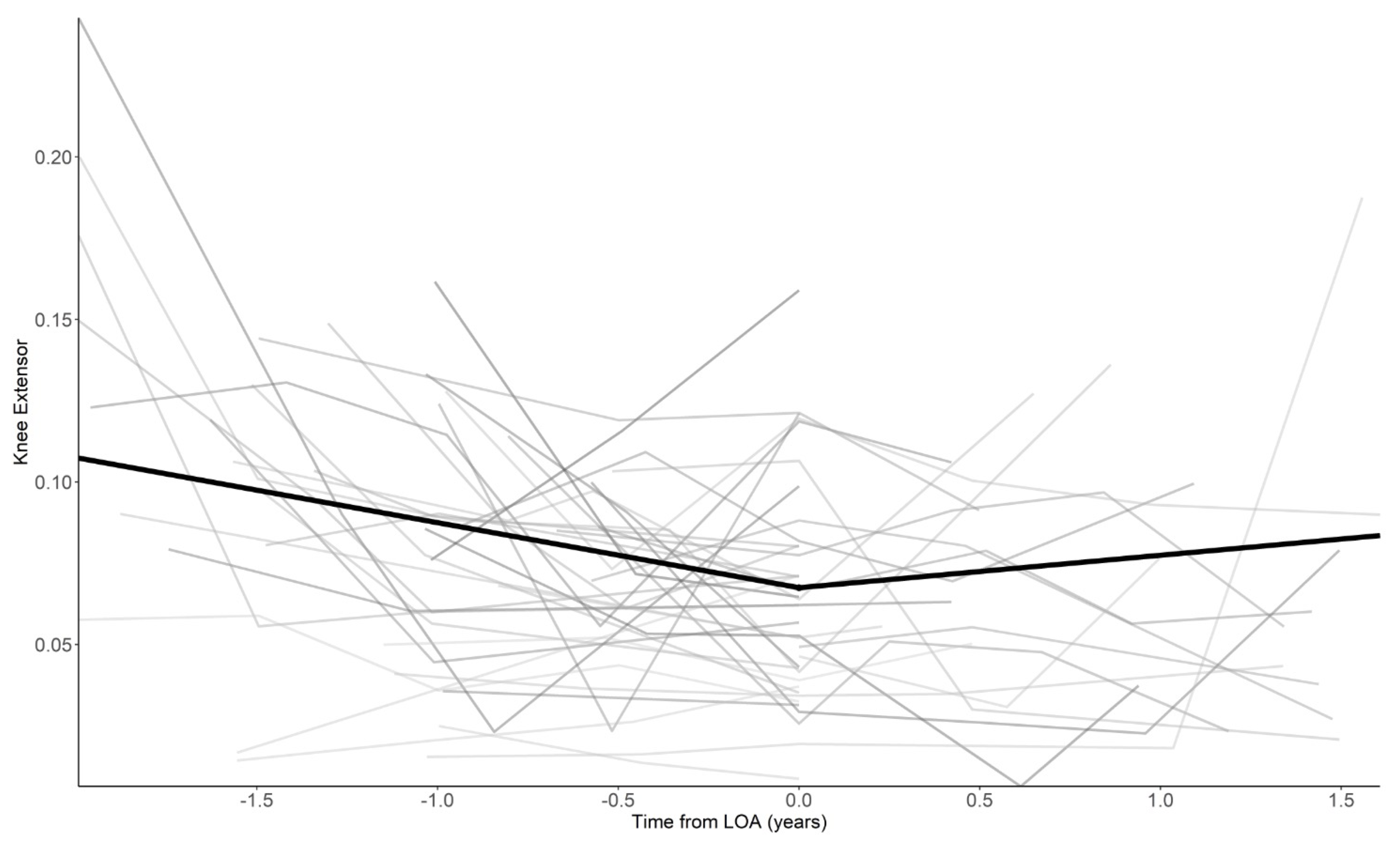

Supplement: S1 Fig — LoA, loss of ambulation. (TIF) [file pone.0304099.s002.tif]
